# Supplementary material for: Mixed-Methods Evaluation of the HealthyWEY E-Learning Toolkit for Promoting Healthy Weight in the Early Years
Source: Int J Environ Res Public Health. 2025 Jan 21;22(2):137. doi: 10.3390/ijerph22020137 (PMC11855305; doi:10.3390/ijerph22020137)
Supplement: Supplementary file 1 [file ijerph-22-00137-s001.zip › Supplementary Resources.pdf]

Supplementary Resource S1. *A breakdown of the 9 modules comprising the HealthyWEY e-learning toolkit, along with their aims and learning objectives.*

**Module**	**Module Aim**	**Learning Objectives**
**1. Communicating with Parents about Child Weight**	• *Equip EYPs with the skills needed to discuss weight issues with parents/carers of infants and young children*	• *Understand the importance of effective communication when discussing child weight with parents/carers and EYPs role in this*<br>• *Use communication strategies to help foster motivation in parents/carers*
**2. Behaviour Change Techniques**	• *Equip EYPS with knowledge and understanding of several behaviour change techniques they can employ in practice*	• *Understand the importance of using behaviour change techniques when working with families of young children*<br>• *Apply appropriate behaviour change techniques to support families to make changes to their physical activity and diet*
**3. Why Weight Matters**	• *Provide an overview of unhealthy weight (both over- and underweight) in the early years and aims to equip EYPs with the key evidence to unhealthy weight in childhood*	• *Describe the prevalence of unhealthy weight at pre-school age*<br>• *Explain the impact of over- and underweight on young children's health (both short and long-term)*
**4. Assessing Weight in Young Children**	• *Equip EYPs with the skills and knowledge to identify unhealthy weight in pre-school children and explain children's weight status to parents*	• *Explain BMI and how BMI centiles are used to diagnose weight issues in children*<br>• *Use BMI charts to identify whether a child has healthy weight, underweight or overweight*
**5. Infant and Child Nutrition**	• *Equip EYPs with knowledge around infant feeding and pre-school nutrition and how to support parents/carers in making changes to their family's dietary behaviours*	• *Describe infant feeding options and their impact on health*<br>• *Discuss the introduction of complementary foods and their impact on healthy eating*<br>• *Support families to develop healthy eating behaviours*
**6. Physical Activity and Sedentary Behaviour**	• *Provide an overview of physical activity and sedentary behaviour and its importance to babies, toddlers and pre-school children – and highlight the types of activity that can be recommended to young children and their families*	• *Explain the importance of physical activity for health*<br>• *Explain the recommended physical activity levels for children <5 years*<br>• *Work with families to identify ways of increasing physical activity and reducing sedentary behaviour*

Supplementary Resource S1 *continued.*

**7. Nutrition, Physical Activity and Weight During Pregnancy**	• *Equip EYPs with knowledge around nutrition and physical activity during pregnancy, optimal gestational weight gain and how best to use this knowledge to support women during their pregnancy*	• *Understand the importance of nutrition and physical activity during pregnancy (for both maternal and child health)*<br>• *Understand optimal weight gain during pregnancy and why it is important*<br>• *Support pregnant women to develop healthy eating and physical activity behaviours*
**8. Cultural Considerations**	• *Equip EYPs with key understanding around the importance of culture in weight-related health*	• *Understand the importance of demonstrating cultural awareness, knowledge and respect*<br>• *Understand the impact of culture on childhood weight, diet and physical activity*
**9. Roles and Responsibilities**	• *Help EYPs reflect on their role in managing child weight, and identify strategies for raising the issue of weight in different situations*	• *Identify the most appropriate support for pre-school children with weight issues and EYPs role within this*<br>• *Identify strategies for raising the issue of child weight in different situations*

Supplementary Resource S2. *Full questionnaire used to assess participants' knowledge, motivations and perceived barriers to addressing pre-school weight.*

HealthyWEY Baseline Questionnaire - Early Years Practitioners

Title of Study: Implementation and evaluation of a child weight e-learning toolkit (HealthyWEY) for maternity, health visiting and children's centre workforces.

As a participant in the HealthyWEY project, Liverpool John Moores University invites you to complete the following questionnaire. This should take approximately 20-30 minutes to complete.

Section 1: During the first part of this questionnaire, you will be asked to provide your demographic details.

Section 2: In the second part of this questionnaire, you will be presented with a series of fictional, practice-based scenarios to consider. After reading these scenarios, you will be asked to respond to a series of questions that will require you to apply your knowledge as an early years professional. **Please note that your responses to these scenarios are in no way an audit of your practice.**

Section 3: In the final section of the questionnaire, you will be asked to respond to a series of statements that will explore:

a) The current challenges and barriers to addressing child weight

b) Your experiences of undertaking activities related to child weight management

c) Your reasons for addressing child weight within your current role

Participant ID

Please provide your participant ID code below: *Required*

HealthyWEY E-Learning Toolkit

Have you completed any of the HealthyWEY e-learning toolkit before?

- Yes
- No

HealthyWEY E-Learning Toolkit

Was this part of:

- ☐ Daisy Bradbury's PhD in Blackburn (2018)
- ☐ Maria Nugent's NIHR internship in Blackburn (2019-2020) Focus
- ☐ groups in the summer of 2021
- ☐ Not sure
- ☐ Other

If you selected Other, please specify:

Which HealthyWEY modules did you complete? (Please tick all that apply)

- ☐ Communicating with parents about child weight Behaviour
- ☐ change techniques
- ☐ Unhealthy weight in pre-school children Nutrition
- ☐ Physical activity and sedentary behaviour
- ☐ Identifying unhealthy weight
- ☐ Culture
- ☐ Roles and responsibilities Can't
- ☐ remember
- ☐

Demographic Details

Please give your age in years

[ ]

Please indicate your gender

- ○ Male
- ○ Female
- ○ Other

Which of the following best describes your ethnic group or background?

- ○ White
- ○ Asian or Asian British Caribbean
- ○ or Black
- ○ Mixed or multiple ethnic group
- ○ African
- ○ Other ethnic group

What is your profession?

[ ]

How many months/years have you worked in this profession?

What is the name of the Trust/Organisation that you work for?

Are you a child weight champion for the HealthyWEY project?

- Yes
- No

Please give details of any prior training you have had around child weight, physical activity, diet or behaviour change

Scenarios

In this section you will be given three scenarios. Please read each scenario carefully and answer the questions about what you would do in that situation.

Below are some notes that will help you to complete this section of the questionnaire

- The scenarios describe some complex situations, so it is important to remember there is not a specific "*right*" or "*wrong*" answer. Your answers may also depend on your professional role.
- Please explain what you would *actually* do, rather than what you *think* needs to happen to resolve the situation (which may not be possible to achieve in your role).
- Please provide as much detail as possible about your reasons for your actions.
- If the individual in the scenario is outside of the age range you usually work with, try and imagine a situation in which you might be in contact with that individual in your professional role (e.g. if you are a midwife, a parent/carer might express concerns about their older child; or if you are a health visitor, a parent/carer of a 2-year old might be pregnant with a second child and ask for your advice).
- If you cannot think of any situation in which these conversations would occur in your professional role, please write "not applicable" and explain why.

Scenario 1 - George

George is 2 years old and lives with his Mum (Tina) and his older brother (Eric, 8 years old). Tina is a single working parent and works shifts at Tesco. When the children aren't at nursery and school, George's Grandma comes round to the house to look after George and Eric. She cooks tea for George and Eric most nights, usually something like fish fingers, oven chips and beans. She gives them both a chocolate biscuit as a treat if they eat their whole meal. After tea, George and Eric usually watch TV until their Mum gets home at 7pm.

Even though George is only 2, he eats as much as Eric and has a Body Mass Index that puts him in the overweight range for his age. At his 2-year check, Tina tells the health visitor she is a bit worried about George, but that his Grandma thinks she's being silly - it's only "puppy fat" and children at 2 are supposed to be "podgy".

Based on the information you have been provided with, what are your initial thoughts about George's diet, physical activity and weight?

What would you do to support Tina in managing George's diet, physical activity and/or weight, and why?

Would you consider onward referral?

○ Yes

- [ ] No

Scenario 1 - George

What are your reasons for onward referral, and who would you refer to?

Scenario 2 - Aalia

Aalia is 4 years old and lives with her Mum (Rani), Dad and four older siblings (aged 8, 10, 12 and 15). Rani comes to have a chat with you because she is worried that Aalia is very skinny compared with her siblings. Rani says Aalia is a good eater though, they have lots of Indian food with lots of vegetables, and Aalia is happy to eat most things. Aalia is also very active and loves to play outside and go to the park.

Based on the information you have been provided with, what are your initial thoughts about Aalia's diet, physical activity and weight?

What would you do to support Rani in managing Aalia's diet, physical activity and/or weight, and why?

Would you consider onward referral?

- Yes
- No

Scenario 2 - Aalia

What are your reasons for onward referral, and who would you refer to?

Scenario 3 - Sophie

Sophie has just found out she is pregnant with her second child. Since having her first child (now 2 years old), she has put on a lot of weight and is now living with obesity.

Sophie has got into unhealthy lifestyle habits and is keen to "sort her health out" as she is worried about the impact her weight and lifestyle might have on her baby. She doesn't currently do *any* physical activity and she admits to eating a very "beige" diet that lacks fruit and vegetables. She doesn't know where to start and wants to ask your advice about what she could do to help the health of her baby.

Based on the information you have been provided with, what are your initial thoughts about Sophie's diet, physical activity and weight?

What would you do to support Sophie in managing her diet, physical activity and/or weight, and why?

Would you consider onward referral?

- Yes
- No

Scenario 3 - Sophie

What are your reasons for onward referral, and who would you refer to?

Barriers to Managing Child Weight

You will now be presented with a series of statements that will explore the current challenges to addressing weight issues in the under 5s.

To what degree do the following barriers prevent you from addressing weight issues in children?

Please don't select more than 1 answer(s) per row.

	1 Not At All A Barrier	2	3	4	5	6 Very Much A Barrier
1. Being unfamiliar with referral routes and pathways	☐	☐	☐	☐	☐	☐
2. Being unfamiliar with services to which to refer a child and their family	☐	☐	☐	☐	☐	☐
3. I don't see addressing child weight issues as being within my job responsibilities	☐	☐	☐	☐	☐	☐
4. Not having resources on child weight-related health such as hand-outs to share with families	☐	☐	☐	☐	☐	☐
5. Not having the time to address a child's weight	☐	☐	☐	☐	☐	☐

To what degree do the following barriers prevent you from addressing weight issues in children?

Please don't select more than 1 answer(s) per row.

	1 Not At All A Barrier	2	3	4	5	6 Very Much A Barrier
6. Not having knowledge of child weight-related health topics such as nutrition and physical activity	☐	☐	☐	☐	☐	☐
7. There are often more pressing issues that I need to deal with than a child's weight	☐	☐	☐	☐	☐	☐
8. Not knowing how to raise a child's weight-related issue with parents/carers	☐	☐	☐	☐	☐	☐
9. Families not perceiving their child's weight to be a problem	☐	☐	☐	☐	☐	☐
10. Worrying about a families' reaction if I initiate a discussion about their child's weight	☐	☐	☐	☐	☐	☐

To what degree do the following barriers prevent you from addressing weight issues in children?

Please don't select more than 1 answer(s) per row.

	1 Not At All A Barrier	2	3	4	5	6 Very Much A Barrier
11. Not feeling prepared to address families' reactions to discussing their child's health	☐	☐	☐	☐	☐	☐
12. Families not supporting my efforts to address their child's weight-related health	☐	☐	☐	☐	☐	☐
13. Families with limited resources (e.g. money, time, capacity) not being able to follow through with weight-related recommendations	☐	☐	☐	☐	☐	☐
14. Worrying that addressing a child's weight-related health would harm my relationship with parents/carers	☐	☐	☐	☐	☐	☐
15. Not feeling confident in discussing a child's weight-related health with their families	☐	☐	☐	☐	☐	☐

To what degree do the following barriers prevent you from addressing weight issues

in children?

Please don't select more than 1 answer(s) per row.

	1 Not At All A Barrier	2	3	4	5	6 Very Much A Barrier
16. I feel it is often an inappropriate time to address a child's weight-related health	☐	☐	☐	☐	☐	☐
17. Being unfamiliar with cultural practices and attitudes towards weight which are different from my own	☐	☐	☐	☐	☐	☐
18. Being unfamiliar with the traditional foods of different cultures	☐	☐	☐	☐	☐	☐
19. Parents not having fluency in English can prevent me from discussing a child's weight-related health	☐	☐	☐	☐	☐	☐
20. I find current weight measurement tools (i.e. growth charts and BMI) are not suitable for all ethnic groups	☐	☐	☐	☐	☐	☐

Feelings About Managing Child Weight

The following statements represent feelings professionals have in relation to managing weight in the under 5s.

Please answer the following questions by considering how you typically feel in relation to child weight management (under 5s)

Please don't select more than 1 answer(s) per row.

	1 False	2 Mostly False	3 More False Than True	4 More True Than False	5 Mostly True	6 True
1. I feel that I am able to engage in child weight-related conversations that are personally challenging	☐	☐	☐	☐	☐	☐
2. I feel connected to my colleagues who are also involved in managing child weight	☐	☐	☐	☐	☐	☐
3. I feel like I can talk to my colleagues about child weight management	☐	☐	☐	☐	☐	☐
			☐	☐	☐	☐

4. I feel confident I can do what is required to manage child weight	☐	☐				
5. I feel a sense of connection with my colleagues because we face the same challenges with child weight management	☐	☐	☐	☐	☐	☐
6. I feel confident in my ability to interpret and talk to parents about child BMI	☐	☐	☐	☐	☐	☐

Please answer the following questions by considering how you typically feel in relation to child weight management (under 5s)

Please don't select more than 1 answer(s) per row.

	1 False	2 Mostly False	3 More False Than True	4 More True Than False	5 Mostly True	6 True
7. I feel close to my colleagues who appreciate how difficult child weight management can be	☐	☐	☐	☐	☐	☐
			☐	☐	☐	☐

8. I feel free to make professional judgements about child weight management	☐	☐				
9. I feel free to make my own decisions when working with families regarding child weight	☐	☐	☐	☐	☐	☐
10. I feel capable of using behaviour change techniques to support parents/carers and children	☐	☐	☐	☐	☐	☐
11. I feel like I am in charge of the activities I do to manage child weight	☐	☐	☐	☐	☐	☐
12. I feel like I am capable of talking about diet with parents/carers	☐	☐	☐	☐	☐	☐

Please answer the following questions by considering how you typically feel in relation to child weight management (under 5s)

Please don't select more than 1 answer(s) per row.

	1 False	2 Mostly False	3 More False Than True	4 More True Than False	5 Mostly True	6 True
			☐	☐	☐	☐

13. I feel like I have a say in choosing how to manage child weight	☐	☐				
14. I feel connected to the colleagues I interact with about child weight issues	☐	☐	☐	☐	☐	☐
15. I feel like I am capable of talking about physical activity with parents/carers	☐	☐	☐	☐	☐	☐
16. I feel like I get along well with colleagues who also work in child weight management	☐	☐	☐	☐	☐	☐
17. I feel free to choose how I support families to manage children's weight	☐	☐	☐	☐	☐	☐
18. I feel like I am the one who decides the best approach to take with each family	☐	☐	☐	☐	☐	☐

Motivations Related to Child Weight

The following question relates to the reasons *why* you would address child weight issues (under 5s) within your role.

The reason I would address child weight issues in my role is...

Please don't select more than 1 answer(s) per row.

	1 Not True At All	2	3	4 Somewhat True	5	6	7 Very True
1. Because I feel that I want to take responsibility for helping children with their weight	☐	☐	☐	☐	☐	☐	☐
2. Because I would feel guilty or ashamed of myself if I did not address child weight within my role	☐	☐	☐	☐	☐	☐	☐
3. Because I personally believe child weight is an important issue	☐	☐	☐	☐	☐	☐	☐

4. Because I would be accountable to others if I did not	☐	☐	☐	☐	☐	☐	☐
5. I really don't think about it	☐	☐	☐	☐	☐	☐	☐

The reason I would address child weight issues in my role is...

Please don't select more than 1 answer(s) per row.

	1 Not True At All	2	3	4 Somewhat True	5	6	7 Very True
6. Because I have carefully thought about it and believe it is very important to address child weight within my role	☐	☐	☐	☐	☐	☐	☐
7. Because I would feel bad about myself if I did not address child weight issues	☐	☐	☐	☐	☐	☐	☐

8. Because it is an important choice I really want to make	☐	☐	☐	☐	☐	☐	☐
9. Because I feel pressure from others to do so	☐	☐	☐	☐	☐	☐	☐
10. Because it is easier to do what I am told rather than think about it	☐	☐	☐	☐	☐	☐	☐

The reason I would address child weight issues in my role is...

Please don't select more than 1 answer(s) per row.

	1 Not True At All	2	3	4 Somewhat True	5	6	7 Very True
11. Because it is consistent with my personal values	☐	☐	☐	☐	☐	☐	☐
12. Because I want others to approve of me	☐	☐	☐	☐	☐	☐	☐

13. Because it is an important component of my role to take responsibility for child weight issues	☐	☐	☐	☐	☐	☐	☐
14. Because I want others to see I can do it	☐	☐	☐	☐	☐	☐	☐
15. I don't really know why	☐	☐	☐	☐	☐	☐	☐

End of Survey

You have now reached the end of this survey.

To submit your answers, please click the '***Finish***' button.

Please note, if you do not click the '*Finish*' button your answers will not be recorded.

Thank you for your participation.

Final Page
